# Pore-Scale Simulation and Sensitivity Analysis of Apparent Gas Permeability in Shale Matrix

**DOI:** 10.3390/ma10020104

**Published:** 2017-01-25

**Authors:** Pengwei Zhang, Liming Hu, Jay N. Meegoda

**Affiliations:** 1 State Key Laboratory of Hydro-Science and Engineering, Department of Hydraulic Engineering, Tsinghua University, Beijing 100084, China; zpw12@mails.tsinghua.edu.cn; 2 Department of Civil and Environmental Engineering, New Jersey Institute of Technology, Newark, NJ 07102, USA; jay.meegoda@njit.edu

**Keywords:** nano-scale gas flow, pore network model, apparent permeability, low connectivity, shale gas

## Abstract

Extremely low permeability due to nano-scale pores is a distinctive feature of gas transport in a shale matrix. The permeability of shale depends on pore pressure, porosity, pore throat size and gas type. The pore network model is a practical way to explain the macro flow behavior of porous media from a microscopic point of view. In this research, gas flow in a shale matrix is simulated using a previously developed three-dimensional pore network model that includes typical bimodal pore size distribution, anisotropy and low connectivity of the pore structure in shale. The apparent gas permeability of shale matrix was calculated under different reservoir pressures corresponding to different gas exploitation stages. Results indicate that gas permeability is strongly related to reservoir gas pressure, and hence the apparent permeability is not a unique value during the shale gas exploitation, and simulations suggested that a constant permeability for continuum-scale simulation is not accurate. Hence, the reservoir pressures of different shale gas exploitations should be considered. In addition, a sensitivity analysis was also performed to determine the contributions to apparent permeability of a shale matrix from petro-physical properties of shale such as pore throat size and porosity. Finally, the impact of connectivity of nano-scale pores on shale gas flux was analyzed. These results would provide an insight into understanding nano/micro scale flows of shale gas in the shale matrix.

## 1. Introduction

The shale pore structure is of great interest in studying gas flows and shale gas extractions. Several researchers have conducted experiments to obtain the shale pore structure and reported that pore spaces in shale (both organic matter and matrix) typically range from several nano-meters to several microns [1,2,3,4,5,6]. In addition, in order to quantify pore structure and its relation to mineralogical grains, pores are classified into three types, i.e., inter-particle pores, intra-particle pores, and intra-particle pores in organic matter [7]. Based on this classification, Mehmani et al. [8,9] developed a multi-scale pore network model to simulate hydrocarbon flow in a shale matrix, which contains both the intra-particle pores in grains and inter-particle pores between grains. Another special feature of pore structure in a shale matrix is the connectivity of pore spaces. Pore space connectivity is determined by the coordination number, which is a generalized mathematical parameter describing how well the pore spaces are inter-connected in porous media. The average coordination number for sandstone is around 4, and the coordination number decreases with the decrease in porosity [10,11,12,13]. For shale matrix, pore connectivity is relatively low [14], and many isolated pores can be observed from two-dimensional SEM images [3,4]. The gas flow conductivity in the shale matrix is mainly determined by the cross-sectional area of pore throats, which is similar in size to the mean free path of gas molecules. Therefore, the gas molecular collision with pore walls tends to be intensified and would cause different gas flow regimes. Hence, gas flow conductivity is not a constant value as in typical macro-porous media [15]. Knudsen number (*K*_n_ = *λ*/*r*) has been defined to differentiate the gas flow regimes, which accounts for the gas molecular mean free path in relation to gas flow characteristic size of porous media [15,16,17]. In particular, observed gas flow regimes varied from viscous flow at low Knudsen number (*K*_n_ < 0.1) to Knudsen diffusion at high Knudsen number (*K*_n_ > 10). During shale gas exploitation, transient release of pore pressure from several tens of MPa to atmospheric pressure would lead to dynamically varying Knudsen numbers. Therefore, single gas flow regime cannot explain the gas flow in shale, and hence multi-flow regimes should be introduced [18,19,20]. A gas flow model considering diffusion and viscous flow was proposed by Song et al. [21]. Since the gas flow regime in shale matrix ranges from slip flow to transition flow [22], Javadpour [19] proposed an apparent permeability to describe gas flow in nano-scale shale matrix by assuming simultaneously occurring slip flow and Knudsen diffusion. Zhang et al. [23] extended the apparent permeability model and proposed the dynamic multi-flow regimes model, which accounted for the coexistence of multi-flow regimes and dynamic variation of different flow regimes. According to all of these theoretical models, the apparent permeability is significantly larger than the intrinsic permeability of shale matrix, especially at lower pore pressures [23,24,25]. In addition, adsorbed phase diffusion also plays an important role in understanding shale gas flow patterns [26]. Researchers have calculated the modified apparent permeability by considering the adsorbed phase transport [27,28,29], and have observed cluster diffusion for adsorbed-phase migration by molecular dynamics simulation [28].

Geometrical structure of shale matrix and flow regimes in nano-scale porous media can pave the way to capturing micro properties of shale gas flow. To simulate fluid transport in micro-scale porous media, computational fluid dynamic methods such as lattice Boltzmann method were successfully applied to explain the multiphase flow mechanisms [30,31]. However, the lattice Boltzmann method is computationally intensive. In contrast, the pore network model is an effective way to capture the fluid transport, and also account the geometrical property of the porous media [32,33]. Recently, a three-dimensional pore network model of a shale matrix, including extracting pore structure from a pack of spheres [8,9] and reconstructing a pore network model using 2D-SEM images [34], was established to simulate gas flow in such complicated porous media. An equivalent three-dimensional pore network model was also developed for a shale matrix, which can capture the porosity, pore size distribution, and connectivity mathematically [35]. In this model, pore bodies and connected throats are regularly shaped, as the capillary force and corner trapped fluid (wetting phase can be easily trapped around the corner of pore structure) are ignored, as it is difficult to quantify such in multi-phase flow simulations.

The main objective of this research is to simulate and analyze the influence of shale gas permeability in continuum-scale using the above model and in situ data. The shale matrix varies with sediment history, which induces a large difference in petro-physical parameters: reservoir pressure, porosity, pore throat size, and the connectivity. Hence, to study the impact of initial reservoir pressure on shale gas exploitation, the shale permeability values were simulated with constant pore throat size as well as throat size obeying normal distribution, especially at low pressure range to show that the apparent permeability of shale varies with possible reservoir pressure variations in shale formations. Then, the sensitivity analysis was performed to determine the contribution to shale permeability from the variation of shale properties such as pore throat size and porosity. Then, generalized equations between apparent permeability and pore throat size or porosity were obtained based on variations on properties of the shale matrix. Finally, the numerical simulations were performed in order to observe the relationship between pore space connectivity and gas flux.

## 2. Shale Matrix Pore Network Model

### 2.1. Petro-Physical Property

**Figure 1:**
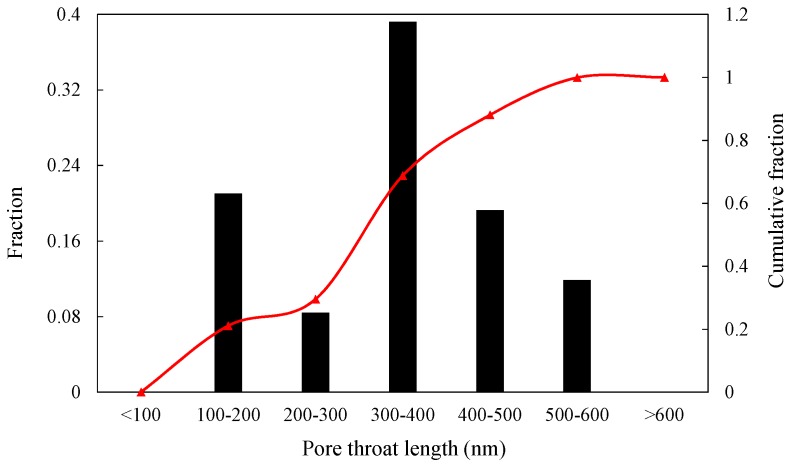
Coordination bond length distribution.

Previous researchers have performed pore size distribution experiments of a shale matrix, and found pore size to be a bimodal distribution [6]. With the known pore size distributions, the total volume of the pore network can be obtained, and then the pore network size can be calculated based on the porosity and the pore center distances. The assumed porosity for this shale matrix is 7%, which is typical for gas bearing shale [5,7]. The average pore diameter was assumed as 300 nm, and pore sizes obeyed the normal distribution and ranged from 50 nm to 500 nm. The pore throat size ranged from 1 nm to 10 nm with the average pore throat diameter of 3 nm. The petro-physical data in this study were based on actual shale data [2,5,7,36,37]. Although the pore center distance was kept a constant value, the length of coordination bond varied due to the size distribution of two adjacent pores. The distribution of coordination bond length and the cumulative fraction are shown in Figure 1.

### 2.2. Pore Connectivity

The coordination number is widely used to mathematically quantify the complicated physical property of pore connectivity. The average coordination number varies for different porous media. An empirical equation (Equation (1)) for calculating average coordination number of the random packing of uniformly sized spheres was proposed by Haughey [38], which depends on porosity of the randomly packed porous media. According to Equation (1), the average coordination number of randomly packed spheres ranged from 2.8 to 12.3 for different packing densities:

(1) $$n_{\mathrm{ave}}=22.47-39.39\varphi ,\;0.259\leq \varphi \leq 0.5,$$

where *n*_ave_ is the average coordination number or the connectivity of porous media, and *φ* is the porosity of the randomly packed sphere.

**Figure 2:**
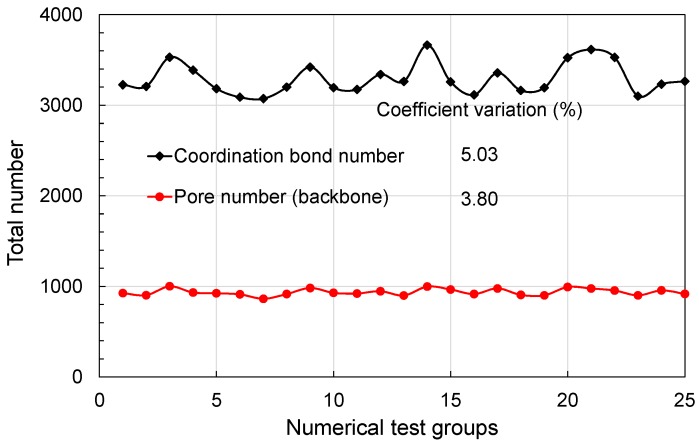
Statistic property of pore number and coordination number.

**Figure 3:**
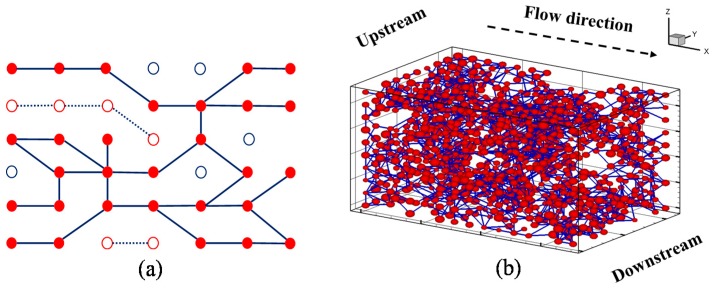
Regular shale matrix pore network model: (**a**) sketch map of diluted pore network; and (**b**) extracted backbone of three-dimensional pore network.

For sandstones, the average coordination number ranges from 3.5 to 4.5 [12], and the coordination number decreases with decrease in porosity [10,12,13]. For typical gas shale, porosity ranges from 1% to 10% [1,5,39], and this number is lower than that for sandstones. Hence, the average coordination number was assumed to be three in this study. The coordination number obeys normal distribution and ranged from 0 to 26, where each pore may have a maximum of 26 possible connections due to the generating method of the regular pore network model [10,40,41,42]. The equivalent pore network model (*n*_x_ = 15, *n*_y_ = 10, *n*_z_ = 10) used here was described in previous research [35]. Generally speaking, the unique properties of this pore network model include the low connectivity and the anisotropy of shale matrix. Figure 2 shows the statistic results, and the minor coefficients of variation showing a good stability–validation for the pore network model. In order to account for the low-connectivity in the shale matrix, dilution procedure, which is similar to the percolation theory, was used [35]. After the dilution procedure, some isolated pores or clusters may emerge (Figure 3a). The backbone of the pore network model, namely pore-structure (interconnected pore bodies) connected with the upstream and downstream boundaries, was extracted for the gas flow simulation, since many isolated pores exist in the shale matrix and do not contribute to the gas flow (Figure 3b). The gas flow direction is along the *x*-axis as shown in Figure 3b. Table 1 shows the difference between the equivalent pore network model (EPNM) used in this research and those of others having typical models of pore-scale analysis of shale gas flow. The major advantage here is that the low-connectivity and anisotropy can be flexibly accounted for and porosity of the whole pore structure is determined.

### 2.3. Pore-Scale Gas Flow Models

Gas flow in typical shale reservoirs falls into slip flow and transition flow range [20]. During transition flow, the slippage effect and Knudsen diffusion occur simultaneously. Hence, the gas flow in shale matrix should consider the slippage effect and Knudsen diffusion [15,20,22,43,44]. Apparent permeability was used to account for the influence of Knudsen number on gas flow regimes [19]. Different apparent permeability models found from literature are summarized in Table 2, where *k*_0_ is the intrinsic permeability that is related to the pore structure of porous media, and it can be obtained by Darcy’s law.

The mathematical model used in this study is similar to the one proposed by Javadpour et al. [19], and slip flow and Knudsen diffusion are considered simultaneously. The only difference is that the Klinkenberg slip flow was introduced instead of Brown’s slip model in Javadpour et al. [19,35]. A detailed explanation was given in Section 3.1. Hence, an equation for mass flux can be expressed as follows [19]:

(2) $$J=-(F\frac{pr^{2}}{8\mu }+\frac{2r}{3}\sqrt{\frac{8RT}{\pi M}})\frac{M}{RT}\nabla p\;,$$

where *R* is the universal gas constant in J/mol/K, *M* is the gas molar mass in kg/mol, *μ* is gas dynamic viscosity in Pa·S, and *F* is the Klinkenberg dimensionless slip coefficient.

During dynamic pore-scale simulation, the mass flux expression can be applied to each pore and coordination bonds. Take pore *i*, for example—the summation of influx and outflux of pore *i* should equal the mass variation during each time step (Equation (3)). Since the pore size is much larger than pore throat size, the pressure drop was assumed to occur only inside the coordination bond. The pressure of coordination bond at each time step equals the average pressure of adjacent pores (Equation (4)):

(3) $$\sum_{j=1}^{n}J_{\mathrm{ij}}=\frac{\partial mi}{\partial t},$$

(4) $${\bar{p}}_{b}=\frac{p_{i}^{k}+p_{j}^{k}}{2},$$

where *J*_ij_ is the mass flux through the bond connected pore *i* and pore *j*, *n* is the number of pores connected to pore *i*, *m*_i_ is the gas mass stored in pore *i*, *p*_b_ is the average pressure of the bond ij, $p_{i}^{k}$ and $p_{j}^{k}$ are the gas pressure of pore *i* and pore *j* at *k* time step, respectively.

The permeability of shale matrix is extremely low, hence the laboratory or in situ tests are time-consuming and expensive to perform [23,45,46]. Here, permeability simulation tests were conducted by using the equivalent pore network model. A pressure difference found in gas bearing shale was applied to the upstream and downstream boundaries. Constant pressures of 20 MPa and 1 MPa were maintained at upstream boundary and downstream boundary, respectively. Other side boundaries were set as no flow. Instead of solving the static mass conservation equation for each pore [8], a dynamic explicit calculation procedure was used. For each time step, this iteration procedure was stopped when the allowable error was reached. The allowable error of pore gas pressure for this simulation was set as one ten-thousandth of downstream boundary pressure (err = *p*_down_/10^4^).

The permeability of this shale matrix based on the pore network model was calculated using the following expression [8]:

(5) $$k_{\mathrm{app}}=\frac{q_{m}\mu l}{A(p_{\mathrm{up}}-p_{\mathrm{down}})\rho_{\mathrm{avg}}},$$

where *q*_m_ is the gas flow rate in kg/s, *ρ*_avg_ is the average gas density through the pore network in kg/m^3^, *A* is the cross section area of the pore network, *p*_up_ is the pressure in the upstream boundary, and *p*_down_ is the pressure at the downstream boundary. Equation (5) is essentially in Darcy’s law, and the only difference is the mass flux term (*q*_m_) accounting for different flow regimes.

## 3. Results and Discussion

### 3.1. Nano-Scale Shale Matrix Gas Flow

**Figure 4:**
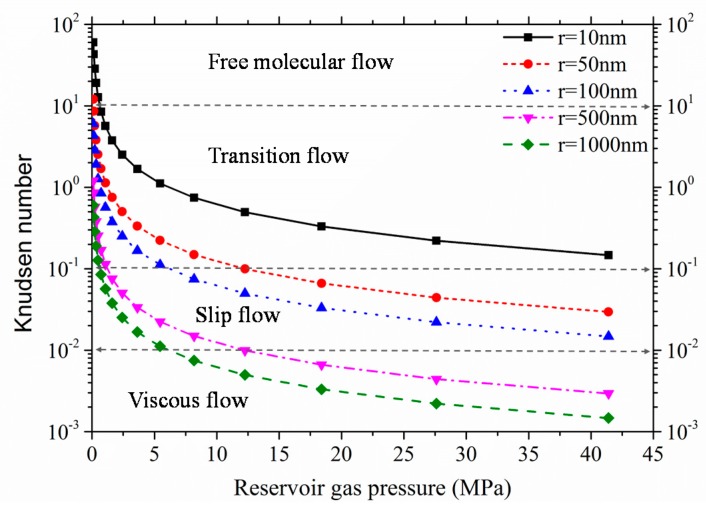
Variation of Knudsen number with reservoir gas pressures.

**Figure 5:**
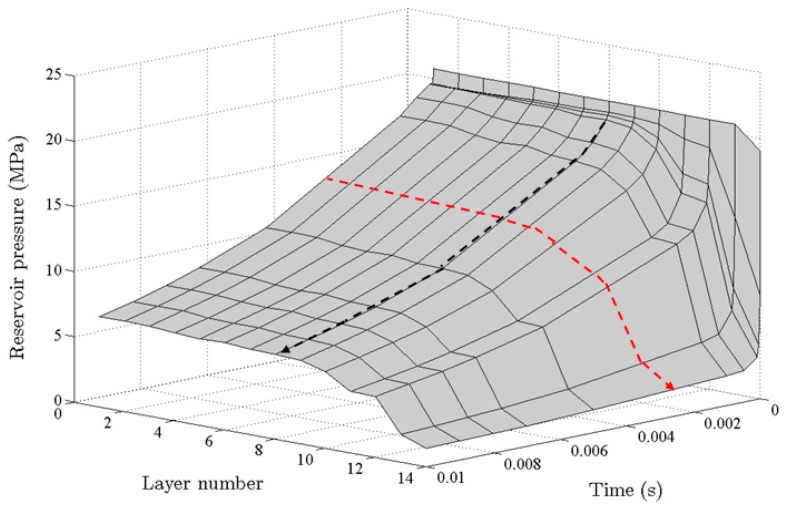
Dynamic gas pressure distribution with time along different layers.

Knudsen number is sensitive to gas pressure and pore throat sizes. Figure 4 shows the variation of Knudsen number with reservoir gas pressure for different pore throat sizes. With the extraction of shale gas (CH_4_ assumed as shale gas), reservoir pressure drops, leading to the increase in Knudsen number. The flow regime varies correspondingly. In addition, when the pore throat size is less than 100 nm, slip flow is the dominant flow regime under typical reservoir gas pressures, which means that the typical pore size for considering slippage effect is about 100 nm. In the proposed pore network model, pore throat size is several nanometers, while the pore size is several hundred nanometers. Therefore, the slip flow and transition flow (includes Knudsen diffusion) are the dominant flow regimes. In order to explain the slippage effect and Knudsen diffusion separately, the Klinkenberg’s slip model was used to couple with Knudsen diffusion instead of Brown’s slip model (Equation (3)). This was because of the rough pore surface considered in the model proposed by Brown [47], and the use of varied tangential momentum accommodation coefficient (α) to account for the roughness of the pore surface. Therefore, the gas molecular collision with pore wall was allowed in this model, and it is not easy to distinguish the dominant flow at different periods. The gas flow in this pore network model is in dynamic equilibrium. The pressure gradient occurs at the downstream layer initially, and then it spreads to the whole pore network gradually. Figure 5 shows a dynamic pressure distribution during gas exploitation, and pore gas pressure at each layer is the average value for a given time step. The dynamic changes of pore pressure will cause the variation of apparent permeability with time.

### 3.2. Apparent Permeability of Shale Matrix

**Figure 6:**
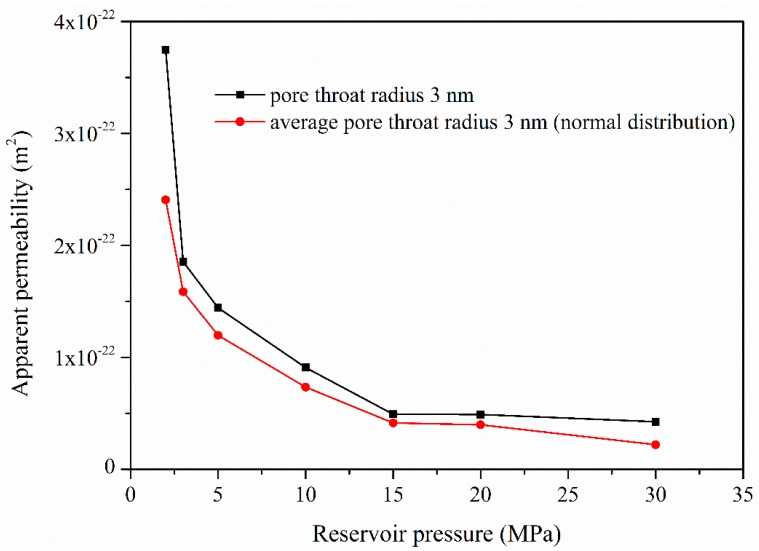
Variation of the apparent gas permeability with reservoir gas pressures.

**Figure 7:**
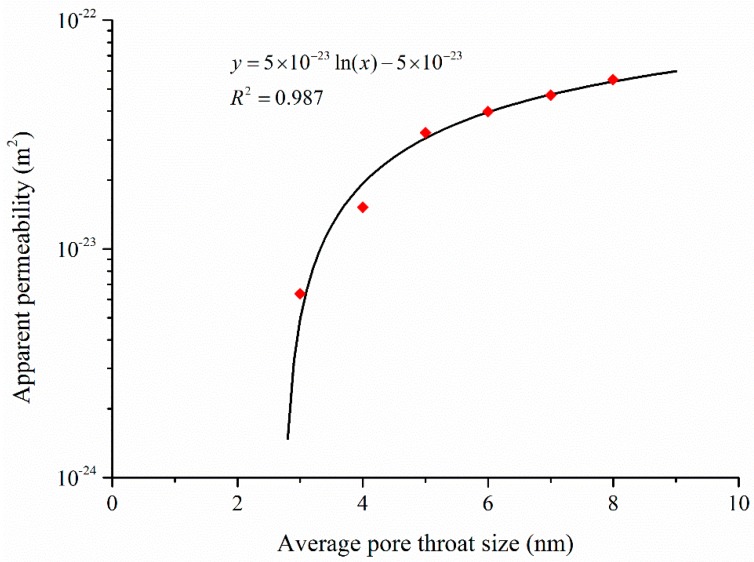
Variation of the apparent gas permeability with pore throat sizes.

Throat size determines the conductivity of flow paths in a shale matrix. Figure 6 shows the apparent gas permeability of shale matrix at different reservoir pressures based on the equivalent pore network model [35]. In this research, two types of pore throat size distributions were compared, specifically constant pore throat size and pore throat size obeying normal distribution. The difference is that reservoir pressure is extended to low pressure range so as to reflect the Knudsen diffusion and slippage effect at the later period of shale gas exploitation. The results indicate that apparent permeability decreases faster in a low pressure range. The apparent permeability is much higher at low pressures due to intensified Knudsen diffusion. Additionally, the results also show that the apparent permeability of constant pore throat size model is larger than that of the one obeying normal distribution, which means that the apparent permeability value of shale strongly relates to the proportion of small pore throats. It is the pore throat size rather than pore size that determines the gas flow regimes, and the sensitivity analysis result of average pore throat size is shown in Figure 7. The result indicates that apparent permeability increases logarithmically with the increases of average pore throat size. The simulated data was fitted to obtain a fitting equation that may provide insights to gas production. Here, the aspect ratio (pore throat size divided by pore body size) is quite small for shale matrix, and hence the pore throat sensitivity analysis was conducted only in a narrow range.

**Figure 8:**
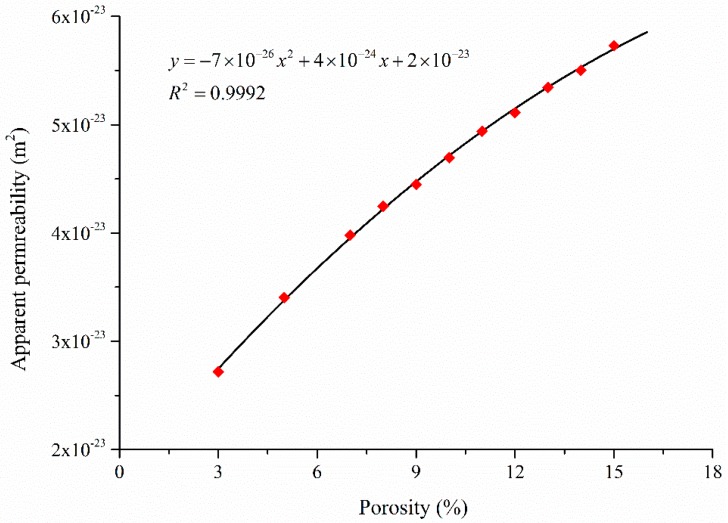
Variation of the apparent gas permeability with porosity.

The porosity of shale varies within a relatively small range, and hence the relation between apparent permeability and porosity (*k*_app_ ~ *φ*) is a useful one. The release of gas pressure during gas extraction causes pores to shrink and the porosity to decrease due to the increase of effective stress. Hence, the apparent permeability is dynamically linked to the shale porosity. Please note that the shale porosity determines the pore center distance. When the porosity increases, the pore center distance decreases. One special case is that once the pore center distance equals the sum of the radii of two adjacent pores (coordination bond disappears), the two adjacent pores are merged into a larger pore. Figure 8 shows that the apparent permeability increases when the porosity increases. The apparent permeability and porosity can be correlated by a quadratic polynomial as shown in Figure 8.

### 3.3. Connectivity in Tight Formations

As mentioned in the previous section, the pore structure of shale matrix is due to the local sediment environment. In this section, the connectivity variation in a two-dimensional pore network model (*n*_x_ = 30, *n*_y_ = 20) was discussed instead of three-dimensional one. Since the variation of connectivity is conducted by changing the average coordination number, the domain size should be enlarged to acquire a stable representative element volume (REV). A three-dimensional pore network model with more than 30 layers in each direction is computationally intense, and hence a two-dimensional pore network model was used. The basic algorithm of establishing the pore network model is the same with that of the three-dimensional one. The coordination number in this simulation varies from three to five, which may represent a certain type of shale or even sandstone.

**Figure 9:**
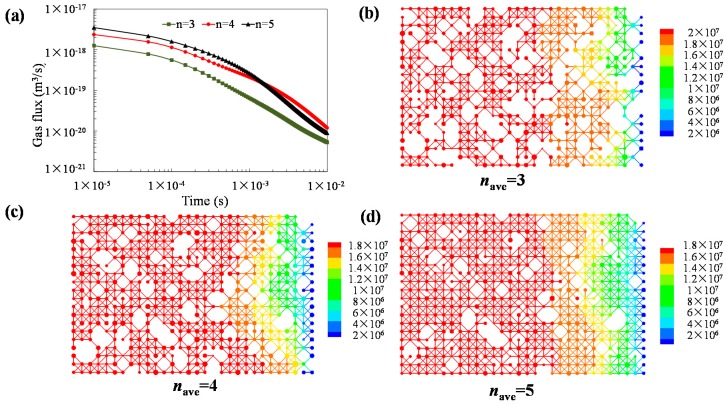
Impact of pore structure connectivity on gas flow in shale matrix: (**a**) gas flux variation; (**b**) pressure contour *n*_ave_ = 3; (**c**) pressure contour *n*_ave_ = 4; and (**d**) pressure contour *n*_ave_ = 5.

Figure 9 shows the dynamic results of gas flows out of shale matrix. As shown in Figure 9a, higher average coordination number means highly connected flow paths, and the gas flux is initially high. There is an intersection of gas flux curves at the later period (Figure 9a), which means that the pressure gradient is lower in the later period for the case of a high coordination number. This phenomenon can be clearly explained by the pressure contours. Higher coordination number means the pressure field in the domain quickly reaches the steady state. Pressure contours in the following figure are showed at the same time, and lower pressure can be found when the equivalent pore network model (EPNM) has higher coordination number. In addition, the preferential flow-path can be detected in the following three contour figures since the pore size is not evenly distributed. We can also detect that, with the increase of average coordination number in EPNM, the preferential flow phenomenon weakens, and the pressure field tends to be evenly distributed (Figure 9b–d). From an application point of view, highly connected shale formation can save the effective gas exploitation time after hydraulic fracturing, since the gas flux varies evenly and it is easier for engineers to optimize the exploitation.

## 4. Conclusions

The nano-scale pore size, extremely low permeability and low pore connectivity are the key features of the shale matrix. The pore network model is a flexible way to simulate gas flow in a tight shale matrix. In this study, a previously developed 3D pore network model was used to investigate the impact of the variation of petro-physical parameters on apparent gas permeability. The following conclusions were drawn based on the simulation results.

Typical gas flow regimes in shale matrix are slip flow and transition flow. The threshold pore size for considering slip flow is roughly 100 nm.

Apparent permeability varies significantly with the reservoir gas pressure, and hence it is not a constant value during shale gas exploitation. The apparent permeability of shale matrix has a logarithmic relation with the average pore throat size, and it is quadratically correlated to the porosity.

Preferential flow weakens with the increase of coordination number, and more even gas distribution or pressure fields were observed at a high coordination number.

Results of these numerical tests may provide meaningful data to better understand micro flow property of shale gas. The above sensitivity analyses of apparent permeability are only valid for micro sections of intact shale, and natural and induced fractures are not considered.

## Figures and Tables

**Table 1:** Comparison of different pore-scale models.

	Mehmani et al. (2013, 2014)	Ma et al. (2014)	Chen et al. (2015)	Present Model
Simulation Method	Pore Network Model	Pore Network Model	LBM (Lattice Boltzmann Method)	Pore Network Model
Constructing pore-scale model	Extract pore network from Finney pack of spheres by Delaunay tessellation method. Finney pack is a dense random pack of identical spheres.	A realistic 3D pore network model of gas shale, and it was constructed from high-resolution 2D grey-scale images. The resolution is 15 nm.	Reconstructed 3D nanoscale porous structures of shale by Markov chain Monte Carlo (MCMC) method based on SEM images of shale samples.	It is a mathematical model, and the pore size and pore throat size distributions are generated based on shale statistic data.
Porosity	Initial porosity is relatively high, shrink some pores and pore throats radii until reaching the porosity of 10% for shale.	2.9%.	Four samples: 19.1%, 22.6%, 26.8%, 17.6%, respectively.	The porosity is 7% assumed in this work according to typical shale data but can be varied based on shale formation. This pore network model is porosity-determined, and it is flexible. Coordination bond length can be calculated by porosity.
Coordination number	Single scale network: average number is 4. Dual scale network (series and parallel).	Less than 3.	Connected with neighboring 18 cells (D3Q19 lattice model).	Average coordination number is 3, and it ranges from 0 to 26.
Connectivity	A fraction of the removed throats (*f*_r_) defined according to the whole pore network model percolation.	Low connectivity.	High connectivity (four samples: 98.0%, 99.1%, 99.7%, and 99.8%).	Each bond has the existing probability, and reduction factor determines the status of the bond open or block.

**Table 2:** Mathematical models of apparent permeability.

Klinkenberg (1941)	$k_{\mathrm{app}}=k_{0}(1+\frac{4\bar{\lambda }}{r})$
Brown et al. (1946)	$k_{\mathrm{app}}=k_{0}(1+{\left(\frac{8\pi RT}{M}\right)}^{0.5}\frac{u}{p_{\mathrm{avg}}r}(\frac{2}{\alpha }-1))$
Beskok and Karniadakis (1999)	$k_{\mathrm{app}}=k_{0}(1+\alpha (K_{n})K_{n}(1+\frac{4K_{n}}{1+K_{n}}))$
	$\alpha (K_{n})=\alpha_{0}\frac{2}{\pi }\tan^{-1}(4{K_{n}}^{0.4})$
Florence et al. (2007)	$k_{\mathrm{app}}=k_{0}(1+\alpha (K_{\mathrm{np}})K_{\mathrm{np}}(1+\frac{4K_{\mathrm{np}}}{1+K_{\mathrm{np}}}))$
	$\alpha (K_{n})=\frac{128}{15\pi^{2}}\tan^{-1}(4{K_{n}}^{0.4})$
Civan (2009)	$k_{\mathrm{app}}=k_{0}(1+\alpha (K_{n})K_{n}(1+\frac{4K_{n}}{1+K_{n}}))$
	$\frac{\alpha_{0}}{\alpha (K_{n})}-1=\frac{A}{{K_{n}}^{B}},\;(A>0,\;B>0)$
